# Expression Patterns of ERF Genes Underlying Abiotic Stresses in Di-Haploid *Populus simonii* × *P. nigra*

**DOI:** 10.1155/2014/745091

**Published:** 2014-03-04

**Authors:** Shengji Wang, Wenjing Yao, Hairong Wei, Tingbo Jiang, Boru Zhou

**Affiliations:** ^1^State Key Laboratory of Forest Genetics and Tree Breeding, Northeast Forestry University, 51 Hexing Road, Harbin 150040, China; ^2^Biotechnology Research Center, School of Forest Resources and Environmental Science, Michigan Technological University, Houghton, MI 49931, USA

## Abstract

176 ERF genes from *Populus* were identified by bioinformatics analysis, 13 of these in di-haploid *Populus simonii* × *P. nigra* were investigate by real-time RT-PCR, the results demonstrated that 13 ERF genes were highly responsive to salt stress, drought stress and ABA treatment, and all were expressed in root, stem, and leaf tissues, whereas their expression levels were markedly different in the various tissues. In roots, *PthERF99*, *110*, *119*, and *168* were primarily downregulated under drought and ABA treatment but were specifically upregulated under high salt condition. Interestingly, in poplar stems, all ERF genes showed the similar trends in expression in response to NaCl stress, drought stress, and ABA treatment, indicating that they may not play either specific or unique roles in stems in abiotic stress responses. In poplar leaves, *PthERF168* was highly induced by ABA treatment, but was suppressed by high salinity and drought stresses, implying that *PthERF168* participated in the ABA signaling pathway. The results of this study indicated that ERF genes could play essential but distinct roles in various plant tissues in response to different environment cues and hormonal treatment.

## 1. Introduction

AP2/ERFs (ethylene response factor) are plants-specific transcription factor family that was firstly identified from tobacco as the binding proteins for reduced sensitivity to disease. AP2/ERFs can be divided into multiple subtribes that include but are not limited to AP2, RAV, ERF, and DREB, according to the difference of their conserved protein domains [1]. In recent years, researches on AP2/ERF transcription factors in various species have shown that AP2/ERFs play an important role in regulating ethylene and drought responsive genes [2–7]. AP2/ERFs contain at least one AP2 conservative domain, which is made up of about 60 highly conserved amino acids [8]. AP2 domain can directly interact with DRE/CRT cis-acting element or GCC-box cis-acting element, and these elements often present the promoter regions of many stress response and tolerance genes [2, 9]. Through regulating the expression of many target genes, ERF family becomes the central part in plant signal transduction network. Most of the members of the ERF family proteins are directly induced. The hormones and environmental cues that can induce ERFs expression include but are not limited to ethylene, jasmine, ABA, salicylic acid, drought, salinity, and cold [10–13]. Interestingly, the same ERF transcription factor can be induced by multiple stress and serves as the converged point of stress-responsive signal transduction pathways in plant body [14, 15]. All these implicate that ERF transcription factors play a key role in plant stress responsive. Further understanding of how and where each ERF functions demands detailed knowledge of spatial-temporal gene expression patterns in response to various abiotic stresses. However, little is known about the tissue-specific and temporal expression patterns of ERFs in response to different abiotic stresses.

In recent years, along with the environment deterioration, high salinity, drought, and low temperature have become the major abiotic factors. However, due to the complexity of interactions and regulation in plants that are subjected to the abiotic stresses, our understanding of the molecular mechanisms of stress tolerance in plants is still very limited. Knowledge of how plants perceive environment cues and then perform signal transduction that lead to augmented stress tolerance is essential for improving plants stress tolerance. Although poplars grow fast in various environments and have been used for timberland, protection forest, and afforestation, poplar growing on marginal lands is subjected to constant abiotic stresses, leading to serious losses in biomass production. Some of the major abiotic stresses are salt and drought stresses. Since its genome was sequenced in 2004 [16], poplar have been widely utilized as a woody model plant species to study the molecular mechanisms and response to the adversity stress. In this study, we detected 176 ERF genes in di-haploid *Populus simonii × P. nigra* and identified 13 ERF genes that were highly responsive to various abiotic stresses and ABA treatment. Then, we studied the spatial-temporal expression patterns of ERF family using real-time reverse-transcriptase- (RT-) PCR. This study provides further insights into the roles of ERF genes in response to abiotic stress in plants.

## 2. Materials and Methods

### 2.1. Plant Culture and Stress Treatments

Twigs of the plant materials from the same clone of di-haploid *Populus simonii × P. nigra* were harvested. For the reproduction of new brunch and root, the twigs were planted in pots containing water and kept under controlled greenhouse conditions of 60–70% relative humidity, 14 h light/10 h dark, and an average temperature of 25°C. Two-month-old seedlings were then subjected to the following treatments: water (normal growth condition without stress: control), 0.15 M NaCl, 25 mM PEG (polyethylene glycol)-6000, or 50 *μ*M ABA for 3, 6, 9, 12, 24, and 36 h. Young root, secondary stem, and leaf tissues were harvested from six seedlings at every time point during each treatments. The harvested tissue samples from each seedling were pooled, frozen immediately in liquid nitrogen, and stored at −70°C for RNA isolation and real-time reverse-transcriptase PCR analysis.

### 2.2. RNA Extraction and RT-PCR Analysis

Total RNA of each sample was extracted using Column Plant RNAout Kit (TIANDZ Corp., Beijing, China) according to the manufacture's instructions. Quality and quantity of RNA were determined by agarose gel electrophoresis and a NanoDrop 2000c Spectrophotometer (NanoDrop Technologies, Wilmington DE, USA), respectively. Approximately one microgram of total RNA was reverse-transcribed to cDNA in a 20 *μ*L volume using 1 *μ*L of RT Primer Mix as primers, and the procedures of cDNA synthesis were following the PrimeScript RT reagent Kit with gDNA Eraser (TaKaRa Corp., Dalian, China). The synthesized cDNA was diluted to 100 *μ*L with sterile water and used as the template for RT-PCR.

Real-time RT-PCR was performed on an ABI 7500 real-time PCR system (Applied Biosystems). ACT, EF1, and UBQ genes were selected as internal controls to normalize the level of total RNA present in each reaction [17]. The primer sequences for real-time RT-PCR are shown in Table 1. The RT-PCR reactions of 20 *μ*L total volume contained 10 *μ*L of SYBR Premix Ex Taq II (TaKaRa), 0.4 *μ*L of ROX Reference Dye II (TaKaRa), 0.4 *μ*M each of forward and reverse primers, and 2 *μ*L of cDNA template (equivalent to 100 ng of total RNA). RT-PCR procedure and conditions are as follows: 10 min 95°C initial denaturation; 40 × 15 sec 95°C denaturation; 60 sec 60°C primer annealing/elongation. The fluorescence was recorded during the annealing/elongation step in each cycle. A melting curve analysis was performed at the end of each PCR by gradually increasing the temperature from 60 to 95°C while recording the fluorescence. A single peak at melting temperature of the PCR-product confirmed primer specificity. RT-PCR was carried out with three technical repeats for each of three biological repasts per clone/treatment to ensure the reproducibility of the results. Expression levels were calculated from the threshold cycle according to the delta-delta *C*_*T*_ method [17]. Relative gene expression level was calculated as the transcription level under stress conditions divided by the transcription level under normal conditions (i.e., samples from plants grown under normal condition and harvested at the same time). Relative gene expression levels were log_2_ transformed.

### 2.3. Screening of ERF Family Genes from Di-Haploid *Populus simonii* × *P. nigra*

176 *Populus trichocarpa* ERF transcription factors were identified through bioinformatics analysis from the PlantTFDB database [18]. Using RT-PCR analysis, total of 59 genes responding to salt stress were initially screened in leaf tissues of di-haploid *Populus simonii × P. nigra*, which were treated with 150 mM NaCl for 24 h. These genes were analyzed by BLASTX against NR and Swiss-Prot databases [19] to search for similarities. The cut-off value for BLASTX was set to *E* value of 10^−5^, and thus ERF genes with *E* value ≤ 10^−5^ were kept for further analysis.

### 2.4. Phylogenetic Analysis of ERFs Sequences

The ERF genes open reading frames were resolved by the ORF Finder provided by NCBI [20]. Genes with complete ORFs were subjected to further analysis. Multiple sequence alignment was performed with ClustalX Version 2.0 [21]. Phylogenetic trees were constructed using MEGA version 5.0 and the neighbor-joining (NJ) method [22].

## 3. Results

### 3.1. Identification of ERF Genes from Di-Haploid *Populus simonii* × *P. nigra*

**Figure 1:**
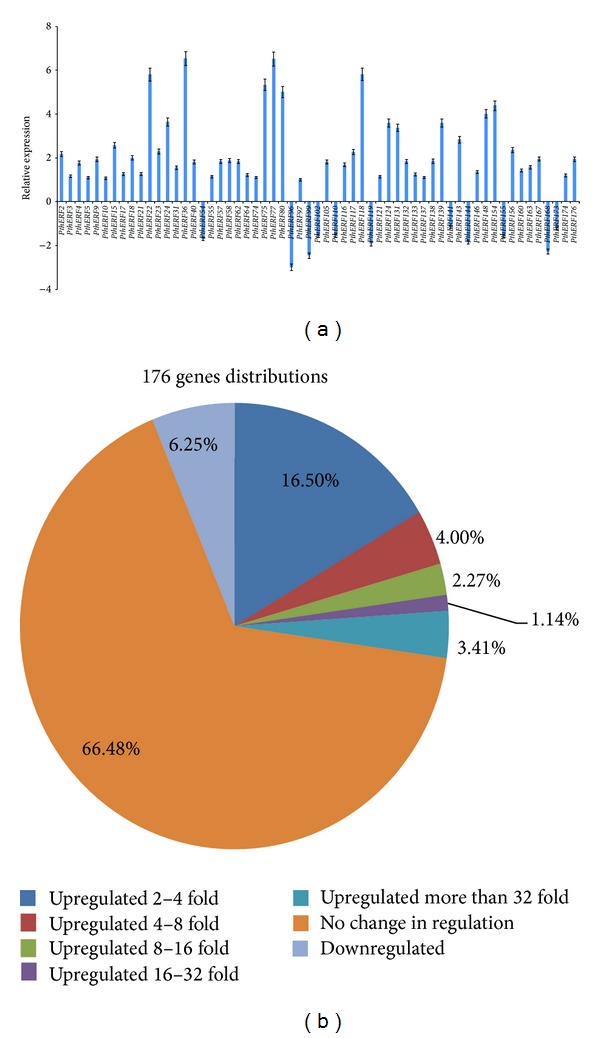
Expression trends of 176 *Populus* ERF genes in response to NaCl stress in the leaves by RT-PCR analysis. Relative expression level was log_2_ transformed: >0: upregulation; =0: no change in regulation; <0: downregulation. (a) Expression patterns of 59 responsive *PthERFs*; (b) 176 ERF genes distributions: 66.48% were no change in regulation; 6.25% were downregulated; the rest were upregulated in different degrees.

**Figure 2:**
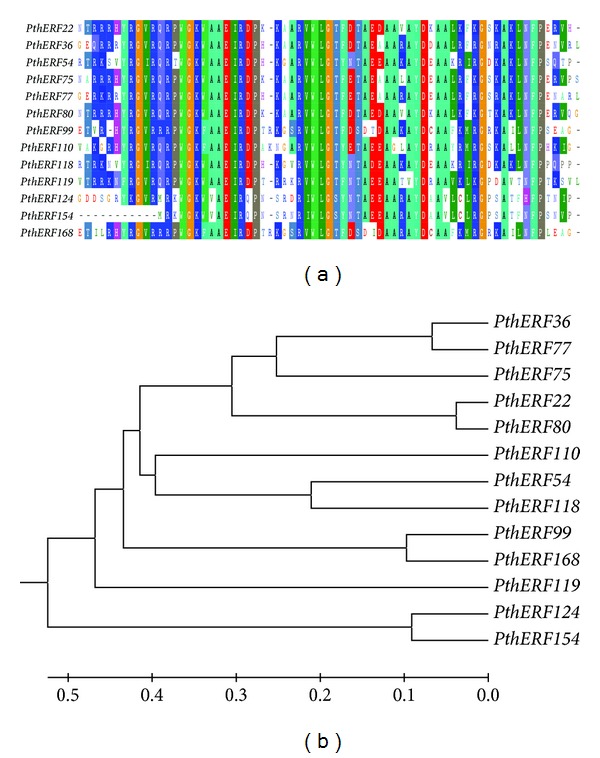
Multiple alignments and phylogenetic analysis of the amino acid sequence of thirteen *PthERFs*. (a) Multiple sequence alignments of 13 *PthERFs* with ERF domain sequences. (b) Phylogenetic analysis of 13 *PthERFs *based on amino acid sequences.

Using RT-PCR, 59 (33.52%) genes responded to high salinity stress in leaves were screened from 176 *Populus* ERF genes, and their expression patterns were displayed in Figure 1(a). Of these 59 genes, 48 (27.27%) were upregulated genes and 11 were (6.25%) downregulated genes. These 176 ERF genes distributions were shown in Figure 1(b). Thirteen ERF genes with full-length ORFs were selected for further study in this research. These genes were designated as *PthERF22*, *36*, *54*, *75*, *77*, *80*, *99*, *110*, *118*, *119*, *124*, *154*, and *168*, and their GenBank accession numbers were shown in Table 1. The ORFs encoded polypeptides of 135–498 amino acids, with MWs between 14.65 and 54.06 kDa and pI values between 4.64 and 9.67 (Table 2). These 13 genes were found to have conserved AP2/ERF domain based on the AP2/ERF domain (59 amino acids) of the tobacco ERF2 protein (Figure 2(a)). The phylogenetic relationships between these ERF genes were deduced from aligned sequences. The phylogenetic tree showed that these thirteen genes could be classified into four subgroups: subgroup 1 contained *PthERF54*, *99*, *110*, *118*, and *168*; subgroup 2 included *PthERF22*, *36*, *75*, *77*, and *80*; subgroup 3 consisted of *PthERF124* and *154*; and subgroup 4 comprised *PthERF119* only (Figure 2(b)).

### 3.2. Relative Abundances of ERFs in Roots, Stems, and Leaves

The relative abundance of the thirteen *PthERFs* was determined by calculating *C*_*T*_ values for each *PthERF* in the leaves, stems, and roots under normal growth conditions according to real-time RT-PCR. *PthERF75* with the lowest expression level in stems (highest delta-delta *C*_*T*_ value) was used as a calibrator (designated as 1.0) to determine relative gene expression levels. Relative gene expression levels were log_2_ transformed and the results are shown in Table 3. There were notable differences in the abundance of these *PthERFs* expression in each tissue, particularly in the stems. The greatest differences among these *PthERFs* in transcript abundances when exposed to normal conditions were 113.7-fold in leaves in *PthERF110*, 702.6-fold in stems in *PthERF118*, and 3.76-fold in roots in *PthERF54*. *PthERF75* was the gene of lowest expression level in leaves and stems, while *PthERF168* was lowest in roots.

### 3.3. Expression Patterns of ERFs in Response to NaCl Stress

**Figure 3:**
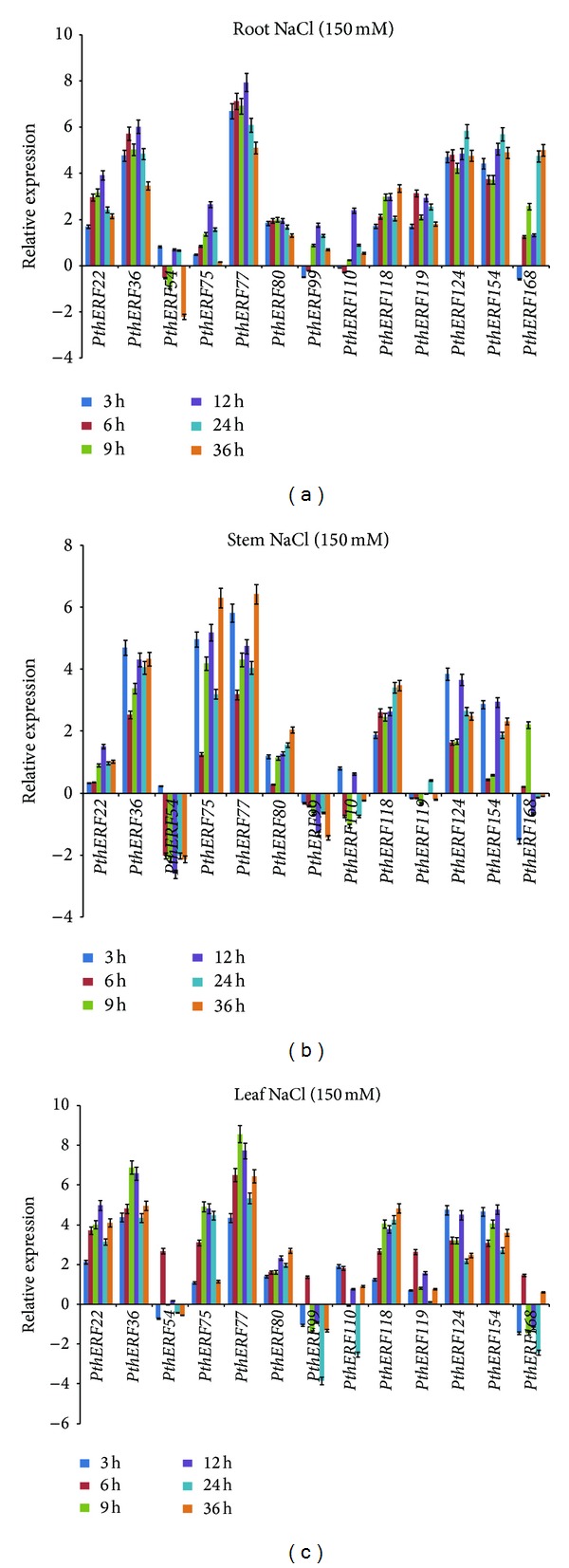
Spatial-temporal expression of 13 *PthERFs* under salt stress in different tissues. Relative expression level was log_2_ transformed: >0: upregulation; =0: no change in regulation; <0: downregulation; (a) in roots; (b) in stems; (c) in leaves.

In roots, all the* PthERFs *(except *PthERF99*, *110*, and *168*) were highly upregulated at 3 h of NaCl stress, but then *PthERF54* showed different expression pattern to other ERF genes at 6, 9, and 36 h. At 12 h, thirteen *PthERFs* reached or nearly reached their highest expression levels in roots, suggesting that *PthERFs* play important roles in salinity stress tolerance in roots (Figure 3(a)). In stem tissues, *PthERF20*, *36*, *75*, *77*, *80*, *118*, *124*, and *154* displayed similar expression pattern, and they were upregulated and highly expressed at each time point. *PthERF119* was downregulated after 3 h of NaCl stress, but the expression of this gene was not significantly different from expression in the control stems during the treatment period. *PthERF168* was significantly downregulated at 3 h of NaCl stress but was induced at 9 h. *PthERF54*, *PthERF99*, and *PthERF110 *were generally downregulated under NaCl treatment (Figure 3(b)). In leaves, the expression patterns of these thirteen ERF genes could be divided into two groups. One group contained *PthERF54*, *99*, and *168*, which were mainly downregulated but highly induced at 6 h. The other group genes were induced except *PthERF110*, which was downregulated at 24 h (Figure 3(c)). Interestingly, all of the 13* PthERFs* were highly induced in leaves at 6 h.

### 3.4. Expression Patterns of ERFs in Response to Drought Stress

**Figure 4:**
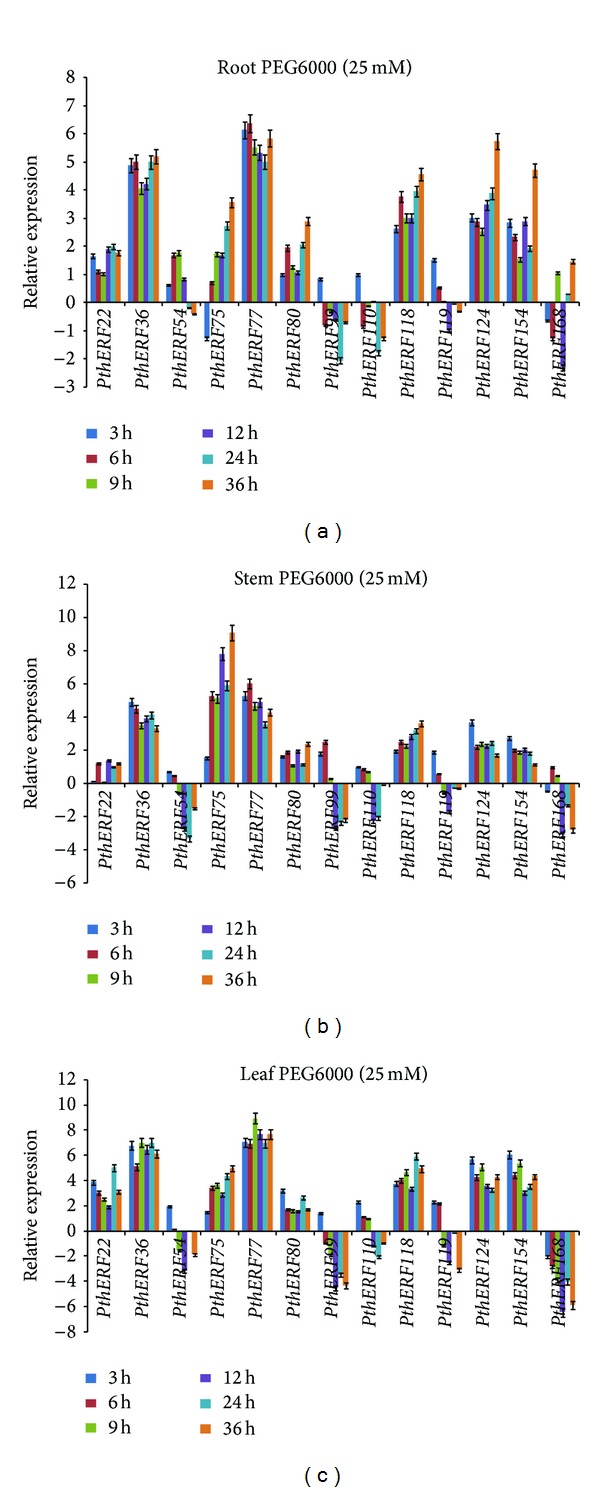
Spatial-temporal expression of 13 *PthERFs* under drought stress in different tissues. Relative expression level was log_2_ transformed: >0, upregulation; =0, no change in regulation; <0, downregulation: (a) in roots; (b) in stems; (c) in leaves.

In roots, all *PthERF* genes were significantly differentially regulated in response to drought stress. They were all induced at 3 h, except *PthERF75* and *PthERF168*. *PthERF99* and *PthERF110* were downregulated by drought stress at the other time points, and other genes were generally upregulated. Specially,* PthERF77* was significantly upregulated during the drought treatment period and reached its highest level at 6 h. *PthERF168* showed different pattern to other genes, and it was highly downregulated at 12 h, but the express did not differ notably from the controls at other time points (Figure 4(a)). In stems, all *PthERF* genes (except *PthERF168*) were induced at 3 h. *PthERF54*, *99*, *110*, *119*, and *168* were significantly downregulated at 12 h. The other genes generally showed similar expression patterns in which the expression levels in leaves were not significantly different during the drought treatment (Figure 4(b)). In leaves, *PthERF168 *was significantly downregulated under drought stress and decreased to its lowest level at 12 h. Other *PthERFs* were induced at 3 h, and eight genes were upregulated at all stress times. *PthERF54*, *99*, *110*, and *119* shared the similar expression pattern, and they were induced at early time points but downregulated at late stages. The results indicated that all these thirteen *PthERFs* play roles in the drought stress response in leaf tissue (Figure 4(c)).

### 3.5. Expression Patterns of ERFs in Response to ABA

**Figure 5:**
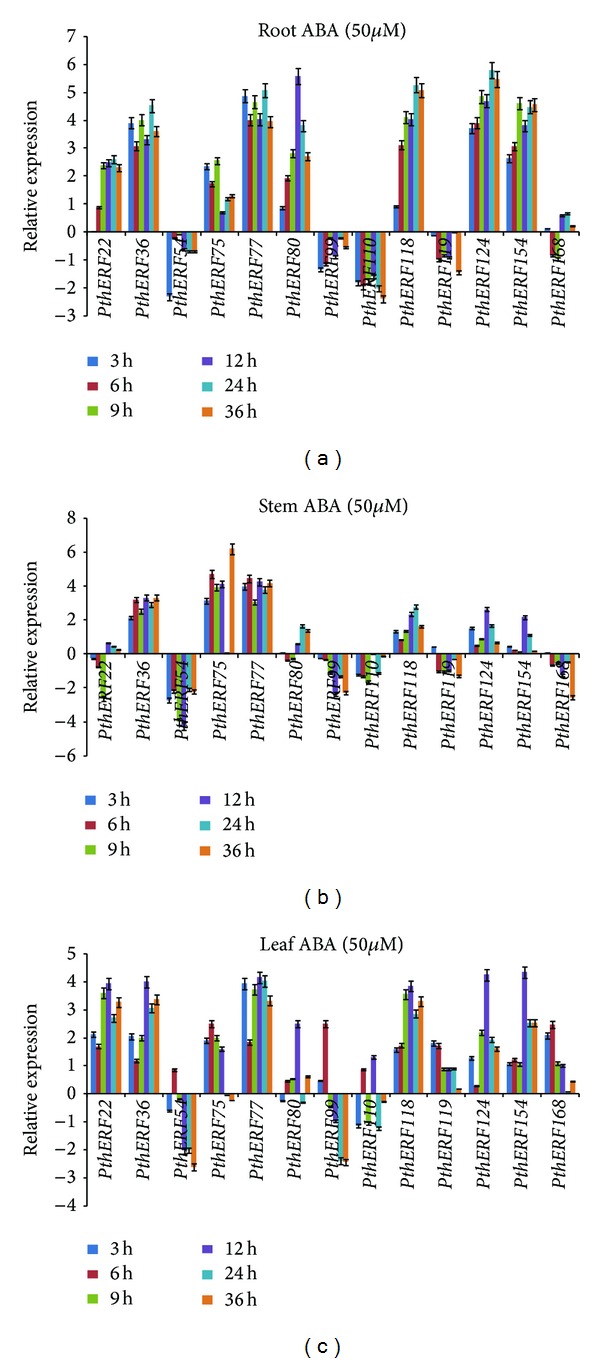
Spatial-temporal expression of 13 *PthERFs* under ABA treatment in different tissues. Relative expression level was log_2_ transformed: >0: upregulation; =0: no change in regulation; <0: downregulation; (a) in roots; (b) in stems; (c) in leaves.

In roots, all *PthERF* genes generally reached their peaks in expression level at 24 h. *PthERF22*, *36*, *75*, *77*, *80*, *118*, *124*, and* 154* were all highly induced during the treatment period, and* PthERF54*, *99*, *110*, and *119* were downregulated. *PthERF168* was downregulated at 6 h and 9 h and upregulated at the other time points, but it did not differ significantly from controls during the whole ABA stress treatment (Figure 5(a)). In stems, *PthERF36*, *75*, and *77* were highly induced by ABA treatment, but *PthERF77* did not show discrepancy from controls at 24 h. *PthERF80*, *118*, *124*, and *154 *showed similar expression patterns, and these genes were generally upregulated or did not differ significantly from the controls. The expressions of *PthERF22*, *54*, *99*, *110*, *119*, and *168* were generally downregulated throughout the treatment period (Figure 5(b)). In leaves,* PthERF54*, *99*, and *110* were induced and generally reach the highest level at 6 h. At the other time points, *PthERF54* and *PthERF99* were significantly downregulated or were similar to the controls. *PthERF110* was upregulated by ABA treatment at 6 h and 12 h. The highest expression levels of the remaining ten *PthERFs* occurred at 12 h, and these genes were significantly induced or did not differ significantly from the controls during the entire treatment time (Figure 5(c)).

## 4. Discussion

Plants are constantly experiencing various biotic and abiotic stresses. For this reason, they have developed specific signal transduction pathways and other molecular mechanisms that allow them to adapt to a variety of stresses by inducing specific sets of genes [23–25]. ERF proteins belong to one of the largest transcription factor families in plants [26]. Such a fact implicates that ERF proteins have crucial roles in regulating responses to environmental stresses and plant development. In the present study, 13 ERF transcription factor genes from di-haploid *Populus simonii × P. nigra* were detected. Since spatial-temporal expression pattern of a particular gene under a given set of conditions can suggest functionality of gene [27–30], and real-time RT-PCR was used to analyze these *PthERFs* expression patterns in response to salinity, drought, and ABA stresses.

In this study, we found that under normal conditions, the abundances of these *PthERFs* were noticeably different in roots, stems, and leaves (Table 3). Except *PthERF54* and *PthERF168*, all of 13 *PthERFs* were highly expressed in stems as compared to either roots or leaves, especially *PthERF118*, whose expression was the highest in the stems. The expression level of *PthERF118* in stem was 215 times of that in roots and 11.5 times in leaves. Such results suggest that the *PthERFs* may play roles mainly in stems rather than in roots and leaves. Previous studies have implicated that the expression of some ERF genes has tissue specific, such as *Medicago sativa* L. *MsERF8* and rice *OsEATB* [31, 32], which are expressed in root and leaf tissues. Conversely, *PthERF54* and *PthERF168* were most abundant in leaves than in root and stem tissues, indicating that they may mainly function in leaves.

When being subjected to NaCl stress, most *PthERFs* were induced by high salinity and exhibited differential expression patterns (Figure 3), especially *PthERF77*, which was most highly upregulated among these genes in all tissues, roots, stems, and leaves. The results suggest that these *PthERFs* are all involved in the salinity stress response. Consistent with these observations, previous studies have shown that ERF genes regulate salt stress response and tolerance. These include but are not limited to *SodERF3* in sugarcane [33], *OsEREBP1* and *OsEREBP2* in rice [34], *HvRAF* in barley [35], and* IbERF1* and* IbERF2* in sweet potato [36]. All these genes can directly confer salt tolerance to plants. In addition, some ERF genes are known to be responsive to salt, but their exact functions in salt stress response and tolerance remain unknown. For example, the expression of an ERF gene inrice, *EsE1*, was induced by salt stress [37]. In *Solanum lycopersicum var. “Pusa Ruby,”* the ERF gene *SlERF68* was upregulated more than 17-fold during salt stress, and the other gene, *SlERF80*, was upregulated up to 400-fold during salt stress [7]. All above suggest that ERF family genes are involved in the salt stress response and may play important roles in high salinity stress tolerance.

Under drought stress, seven *PthERF* genes exhibit similar expression patterns to those under salt stress (Figure 4). Evidence that REFs can confer increased drought tolerance has been implicated in previous studies. These which include *TSRF1* improve salt and drought tolerance of rice seedlings without growth retardation [38]. *SodERF3* of sugarcane increases drought stress tolerance in tobacco [33]. Transgenic plants overexpressing *OsDERF1* (OE) led to reduced tolerance to drought stress in rice at seedling stage, while knockdown of *OsDERF1* expression conferred enhanced tolerance at seedling and tillering stages [39]. Consistent with the result, a previous study [7] also showed that the expression level of the ERF gene, *SlERF5*, in stems had decreased significantly in comparison to the controls after drought stress treatment, but the overexpressing *SlERF5* transgenic plants showed increased resistance to drought stress. Therefore, the downregulation of *PthERFs* in response to drought stress may also be evidence that they play roles in drought tolerance. In this study, five *PthERF* genes, *PthERF54*, *99*, *110*, *119*, and* 168*, were mainly downregulated in drought stress treatment (Figure 4).

The ABA signaling pathways are reported to comprise signal transducers and transcription factors [40, 41], and ABA plays a pivotal role in a variety of developmental processes and adaptive stress responses to environmental stimuli in plants. Interestingly, our result showed that all of the *PthERFs* were highly induced by ABA treatment in the leaves at 6 h and reached a peak at 6 h or 12 h (Figure 5). Presumably, the time lag is because the signal perception and transduction take time to trigger the genes with distance. The induction of ERF genes will impose regulation on their targets genes, which in turn can augment the stress adaptation and tolerance. When such a process is completed, the expression of ERF will be decreased. Some reports also showed that the transcription factors were highly induced at early stress period and then decreased [42–44]. In addition, most *PthERFs* were highly upregulated by ABA stress, implicating that *PthERFs* may be involved in ABA-dependent stress responses. The expression discrepancy of the same genes in various tissues in response to ABA suggests that ERFs have tissues specificity. For example, *PthERF119* was upregulated responded to ABA treatment in leaves but downregulated in roots.

In general, *PthERF36*, *75*, *77*, *118*, and *124* displayed similar expression pattern in response to abiotic stress treatment, indicating that these five genes may be involved in the same gene expression regulatory networks in response to stresses. Other *PthERFs* displayed different expression patterns in response to stress, suggesting that these genes may be involved in distinct gene regulation pathways.

## 5. Conclusion

In conclusion, 13 *PthERFs* expression patterns have been constructed in different tissues of di-haploid *Populus simonii × P. nigra* in response to salinity stress, drought stress, and ABA treatment. The results showed that *PthERFs* can be induced by salinity, drought, and ABA, indicating that *PthERFs* were involved in salt and drought stress tolerance and are controlled by ABA. Further, these *PthERF* genes were more highly induced by NaCl, PEG, and ABA in roots and leaves than in stems, suggesting that these genes may play roles in stress responses in the roots and leaves but not stems.

## Figures and Tables

**Table 1:** Primers used in real time RT-PCR.

Genes	GenBank number	Forward and reverse primers (5′-3′)	
*PthERF22 *	XM_002297841	ACGTGACCCTAAAAAGGCAGCTC	GTGTCTGGTGCAAATGATGAGGG
*PthERF36 *	XM_002302128	GGCACATTTGATACTGCAGAGGC	GTTGCCTTGGAGATGAGATTGGC
*PthERF54 *	XM_002328584	CAAGCTACACCATCCAAGTCCAG	GCTTCATCATATGCTTTGGCGGC
*PthERF75 *	XM_002318140	TTATGGCTCCTCTAGCTCGTTCC	CTGCTGTTGAGAAGATATCGGCC
*PthERF77 *	XM_002306676	GTCATCTGGTGCAACTGCAACTG	CCAGACTCTTGCTGCTTTGTGTG
*PthERF80 *	XM_002304604	GTTCAAAGGCACCAAGGCTAAGC	TCTGGTGCAAATGATGAAGGGGG
*PthERF99 *	XM_002332658	AAGTTTGCAGCAGAGATCCGTG	GATCTTCTTCTCTTCCTGCCTG
*PthERF110 *	XM_002304556	GCTGGATTGAATGAAGCTGCTG	GCATTCTATAAGCCGCCCTATC
*PthERF118 *	XM_002315454	GGCACTTACAACACAGCTGATG	TCAAAACTAGCCATAGCAGCCG
*PthERF119 *	XM_002325598	AAAAACTTCAGGGGTGTCCGTC	TAGTAGGAAAGTTGGTGACGGC
*PthERF124 *	XM_002324777	GGCGACGTTTCATTTTCCAACG	CCCACTAAAAATCCCCTCCAAG
*PthERF154 *	XM_002326261	GAGGAAGCAGCAAGAGCATATG	GATTCCACAATCCTCTCTGCAG
*PthERF168 *	XM_002299371	TAGCACCCAAGAAACCTGTAGC	GTACCTAACCAAACCCGACTAC
*ACT *	JM986590	ACCCTCCAATCCAGACACTG	TTGCTGACCGTATGAGCAAG
*EF1 *	FN356200	AAGCCATGGGATGATGAGAC	ACTGGAGCCAATTTTGATGC
*UBQ *	FJ438462	CGTGGAGGAATGCAGATTTT	GATCTTGGCCTTCACGTTGT

**Table 2:** Characteristics of the 13 *PthERFs* proteins.

Gene	cDNA length (bp)	Mature protein		
		AA	PI	MW (KDa)
*PthERF22 *	696	231	7.09	25.78
*PthERF36 *	1203	400	7.32	42.80
*PthERF54 *	768	255	8.18	29.21
*PthERF75 *	723	240	9.24	26.48
*PthERF77 *	1497	452	9.47	54.06
*PthERF80 *	561	186	9.67	20.97
*PthERF99 *	705	234	4.76	26.04
*PthERF110 *	780	259	8.10	28.46
*PthERF118 *	747	248	9.42	27.37
*PthERF119 *	966	321	6.39	36.45
*PthERF124 *	468	155	8.05	17.31
*PthERF154 *	408	135	4.93	14.65
*PthERF168 *	714	237	4.64	26.65

**Table 3:** Relative mRNA abundance of ERF genes in different tissues under normal conditions.

Gene	Relative abundance		
	Roots	Stems	Leaves
*PthERF22 *	0.41	30.64	3.78
*PthERF36 *	0.79	26.47	6.97
*PthERF54 *	3.76	41.85	111.22
*PthERF75 *	1.0	1.0	1.0
*PthERF77 *	0.21	7.14	1.84
*PthERF80 *	0.42	26.8	9.09
*PthERF99 *	0.24	42.7	38.01
*PthERF110 *	3.3	276.69	113.67
*PthERF118 *	3.27	702.55	61.23
*PthERF119 *	3.24	329.37	61.82
*PthERF124 *	0.22	83.77	46.88
*PthERF154 *	0.26	125.14	25.31
*PthERF168 *	0.04	60.85	108 .57
